# A rare *PHKA2* variant (p.G991A) identified in a patient with ketotic hypoglycemia

**DOI:** 10.1002/jmd2.12041

**Published:** 2019-05-28

**Authors:** Yasuhiko Ago, Hideo Sugie, Tokiko Fukuda, Hiroki Otsuka, Hideo Sasai, Mina Nakama, Elsayed Abdelkreem, Toshiyuki Fukao

**Affiliations:** ^1^ Department of Pediatrics, Graduate School of Medicine Gifu University Gifu Japan; ^2^ Department of Occupational Therapy Tokoha University Shizuoka Japan; ^3^ Department of Pediatrics Hamamatsu University Hamamatsu Japan; ^4^ Department of Neonatology Gifu Prefectural General Medical Center Gifu Japan; ^5^ Division of Clinical Genetics Gifu University Hospital Gifu Japan; ^6^ Faculty of Medicine, Department of Pediatrics Sohag University Sohag Egypt

**Keywords:** case report, enzyme assay, gene panel, glycogen storage disease type IXa, ketotic hypoglycemia, variant *PHKA2*

## Abstract

We describe the case of a 4‐year‐old boy who suffered from frequent ketotic hypoglycemia (KH) but did not have hepatomegaly or elevated liver enzyme levels. However, the patient was found to have a rare variant in the *PHKA2* gene. To detect the underlying disease in this case, we performed a gene panel analysis covering 59 genes that are involved in fatty acid oxidation, ketone body metabolism and transport, and glycogen storage diseases. We found no reported disease‐causing mutations. However, the p.G991A variant in *PHKA2* was detected. The allele frequency of this variant is 4.57 × 10^−5^ in the population worldwide, but in Japan it is 5.15 × 10^−3^. We suspect that this variant may be a major cause of KH in Japanese patients. We performed an enzyme assay on blood cells from the patient. Although the activity of the current PhK variant was not low, it did exhibit thermal instability and a lower affinity to phosphorylase *b* than the wild type. The patient needed bedtime uncooked cornstarch supplementation from age 5 years until he was 9 years old. The patient's condition improved spontaneously without neurological complications. The clinical course and prognosis in this case are similar to those of glycogen storage disease type IXa, which is also caused by an abnormality of *PHKA2*.

**Synopsis**

The p.G991A variant of *PHKA2* could be a major cause of ketotic hypoglycemia in Japanese patients who do not have hepatomegaly or elevated liver enzymes.

## 1 INTRODUCTION

Ketotic hypoglycemia (KH) is a major cause of hypoglycemia; it is responsible for 30%‐50% of cases of childhood hypoglycemia. However, the exact pathogenesis of KH is unknown. Consequently, a diagnosis of KH can be established only after eliminating other known diseases, including glycogen storage diseases (GSDs). Boys are more susceptible to KH than girls are.^1^ In Japan, the prevalence in boys is twice that in girls.^2^

Glycogen storage disease type IXa (OMIM 306000) is a rare X chromosome‐linked disease. Most patients with GSD type IXa are boys. Patients usually present with fasting hypoglycemia, hepatomegaly, growth retardation, and mild elevation of transaminases. These symptoms disappear over time.

Brown et al.^1^ performed genetic analyses of 164 children (96 boys, 68 girls) who had had two or more episodes of KH (blood glucose level <50 mg/dL). The patients did not have hepatomegaly and did not seem to have GSDs. Nevertheless, variants in *PHKA2* were detected in 10 boys and 2 girls. This finding led us to hypothesize that unidentified *PHKA2* variants may be a major cause of childhood KH, and may explain the different incidences of KH in boys and in girls.

## 2 CASE

A 4‐year‐old boy was referred to our hospital because of recurrent KH. He was born at full term and had a birth weight of 2940 g. At the age of 2 years and 7 months, the patient developed KH for the first time. When he was 4 years old, he suffered from recurrent hypoglycemia approximately every 2 months. The patient's hypoglycemic episodes mostly occurred in the morning within 12 hours from his last meal, even though he ate dinner as usual. In particular, the patient became hypoglycemic during infections. At the age of 4 years and 11 months, the patient was started on a bedtime uncooked cornstarch supplement to prevent nocturnal hypoglycemia. Since then, he has experienced no hypoglycemic episodes.

At the age of 5 years and 4 months, the patient's height was 113.0 cm (+0.9 SD) and his weight was 20.4 kg (+0.7 SD). Fasting for 14.5 hours induced hypoglycemia (Table 1). Endocrinologic data, including thyroid‐stimulating hormone, growth hormone, adrenocorticotropic hormone, free triiodothyronine, free thyroxine, insulin, cortisol, epinephrine, norepinephrine, and dopamine, showed no abnormality. Urinary organic acid analysis and blood acylcarnitine analysis showed nonspecific profiles. During our investigations, the patient exhibited no signs of hepatomegaly, and the concentrations of transaminases in his serum were normal. The patient is currently 9 years old, and his physical and mental development is normal. Bedtime supplementation with uncooked cornstarch was stopped when the patient was aged 9 years and 1 month. The patient's elder sister has experienced no hypoglycemic episodes, and his 6‐year‐old brother has experienced KH only once. As far as we examined, there are no relatives who had experienced recurrent hypoglycemia.

**Table 1:** Results of the fasting test

Fasting time	Blood glucose (mM)	Free fatty acid (μM)	β‐hydroxybutyrate (μM)	Acetoacetate (μM)	Lactate (mM)	Alanine (nmol/mL)
0 h	7.16	196	20	41	0.83	264
14.5 h	2.89	3020	996	445	0.67	122

*Note*: Free fatty acid, β‐hydroxybutyrate, and acetoacetate levels were measured in serum. Lactate was measured after removing protein from the blood.

## 3 METHODS

### 3.1 Gene panel analysis

Using the MiSeq Sequencing System (Illumina, San Diego, CA) at the Kazusa DNA Research Institute, we performed mutation analysis using a DNA panel consisting of 59 genes (including *GYS2*, *PHKA2*, *PHKB*, *PHKG2*, *PYGL*, *ACAT1*, *OXCT1*, and *SLC16A1*) suspected to be involved in fatty acid oxidation, ketone body metabolism and transport, or GSDs.

### 3.2 Enzyme assays

Hemolysate from the patient was prepared by the method of Beutler et al.^3^ Phosphorylase *b* kinase (PhK) activity was measured at pH 6.8 using exogenously applied phosphorylase *b* as a substrate following the method of Lederer with minor modification.^4^ To summarize, 50 μL hemolysate was incubated at 37 °C in a final volume of 160 μL containing 6 mM ATP, 10 mM magnesium acetate, 5.6 units rabbit muscle phosphorylase *b*, 50 mM Tris‐HCl (pH 6.8), and 50 mM β‐glycerophosphate. At various time intervals, 20‐μL samples of the incubation mixture were removed and mixed with 150 μL of a cold solution of 5 mM EDTA, 0.1 M NaF, and 40 mM β‐glycerophosphate (pH 6.8); 20 μL of this mixture was used for the phosphorylase *a* assay. The PhK activity was calculated from the phosphorylase *a* activity. The thermal stability of PhK was studied by incubating the hemolysate at 45 °C for up to 30 minutes.^5^, ^6^, ^7^

The affinities of PhK for phosphorylase *b* and ATP were evaluated by changing the concentration of the specific substrate. Michaelis constants (*K*_m_) were determined by double reciprocal plotting of the substrate concentration and the reaction velocity. The thermal stability and affinity results were compared with those of three adult controls.

## 4 RESULTS

### 4.1 Gene panel analysis

The *PHKA2* variant c.2972G>C (p.G991A) (NM_000292.2) was detected (rs761088614) in the patient. The patient's younger brother did not have this variant, and his mother had this variant in the heterozygous state, which was confirmed by Sanger sequencing. In silico analyses were performed by MutationTaster, PolyPhen‐2, PROVEAN, and SIFT. Each result was disease causing, probably damaging with a score of 0.989, deleterious, and damaging, respectively. Based on the American College of Medical Genetics and Genomics (ACMG) recommendations,^8^ the pathogenicity of this variant meets the criteria of PP3 and PP4.

### 4.2 Enzyme assay

**Figure 1:**
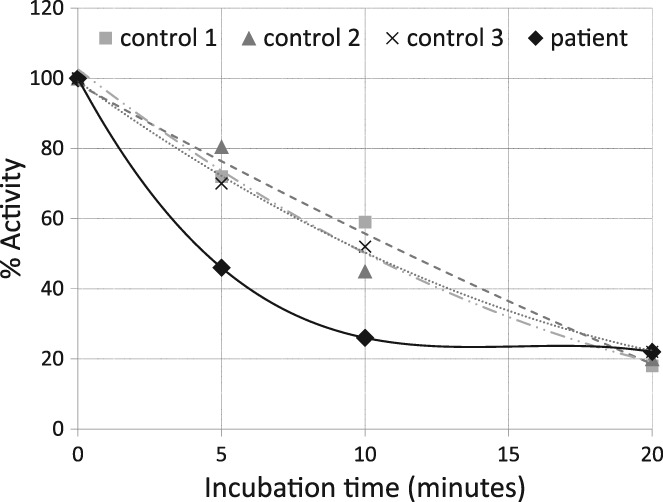
Variant PhK was less stable than putative wild‐type PhK (extracted from erythrocytes of three healthy adults) at a temperature of 45°C. The remaining activity of variant PhK was about 50%‐60% of that of wild‐type PhK after 5‐10 min of incubation

First, we evaluated the activity and the thermal stability of the variant PhK in erythrocytes from the patient. The activity of the variant PhK in the patient's erythrocytes was not significantly different from those of the three controls, as shown in Table 2. However, the variant PhK was less stable than controls at a temperature of 45 °C (Figure 1). Second, we evaluated the affinity of the variant PhK for phosphorylase *b* and ATP. The *K*_m_ values of the patient's PhK with respect to phosphorylase *b* were 75.3 Units/mL, which was higher than those of three controls (mean 35.6 Units/mL, SD 5.84 Units/mL). The *K*_m_ value of the patient's PhK against ATP did not differ from those of controls (data not shown).

**Table 2:** Results of the enzyme activity assay using blood cells

	Control 1	Control 2	Case	Disease control
PhK (nmol/min/gHb)	8.5	13.8	8.1	0.8

## 5 DISCUSSION

We describe the case of a boy who suffered from frequent KH but did not have hepatomegaly or elevated liver enzyme levels. This patient sometimes developed hypoglycemia after overnight fasting. This was confirmed by a fasting test in which the patient developed hypoglycemia after 14.5 hours of fasting. This fasting time was too short to induce ketoacidosis because the total ketone body level was about 1.4 mM, whereas the free fatty acid level was induced to about 3 mM. Consequently, we suspected that the patient might have problems with glycogenolysis rather than with gluconeogenesis, fatty acid metabolism, or ketone body metabolism. The gene panel analysis identified a rare *PHKA2* variant, p.G991A. We considered this variant to be the likely cause of the patient's KH.

Recently, Brown et al.^1^ found that 20 KH patients had variants in genes causing GSDs (including 4 of type 0, 2 of typeVI, 12 of type IXa, 1 of type IXb, and 1 of type IXc) by performing Sanger sequencing on five genes (*GYS2*, *PYGL*, *PHKA2*, *PHKB*, and *PHKG2*) of 164 KH patients without hepatomegaly.^1^ This finding was surprising, because most clinicians do not suspect GSDs unless patients have enlarged livers or present with elevated liver enzyme levels. This unexpected result of Brown et al. led us to consider that the p.G991A variant of *PHKA2* might be the causative factor of KH in our patient. Although the PhK activity in the patient's erythrocyte lysate was within the normal range, we further analyzed the thermal stability of PhK and its *K*_m_. This revealed that the patient's PhK was thermally unstable and had a higher *K*_m_ value than that of the controls.

The p.G991A variant of *PHKA2* is rare. The allele frequency of this variant (rs761088614) is 4.57 × 10^−5^ in the worldwide population (retrieved on November 17, 2018, from: http://exac.broadinstitute.org/variant/X‐18919658‐C‐G). According to a Japanese database (retrieved on November 17, 2018, from: http://www.hgvd.genome.med.kyoto‐u.ac.jp/cgi‐bin/searchBydbsnp.cgi), the allele frequency of this variant in Japan is 5.15 × 10^−3^, which is much higher than that in the global population. This means that about 1 in 200 males in Japan have this variant. The prevalence of KH has not been reported from Japan or from many other countries, but the prevalence of this variant in Japanese males likely reflects the prevalence of KH in Japan. If KH is caused by a *PHKA2* variant in most patients, the treatment strategy for such KH patients should be similar to the treatment for GSD type IXa.

One limitation of the current study is that it concerns a single case only, and our experience is still limited. However, we carried out some preliminary analyses of the presence of the p.G991A variant in KH patients in Japan and found that several KH patients did possess the variant (unpublished data, Fukao et al.). Further research will be necessary to investigate whether this variant is the most important genetic factor causing KH in Japan.

## 6 CONCLUSION

A rare p.G991A variant of *PHKA2* was identified in a Japanese KH patient. The variant resulted in some functional abnormalities and may have been the cause of KH in this patient.

## CONFLICT OF INTEREST

The authors declare no potential conflict of interest.

## AUTHOR CONTRIBUTIONS

T.F. and Y.A. were involved in clinical management of the patient. H.S., H.O., M.N., and E.A. were involved in gene panel analysis and Sanger sequencing. H.S. and T.F. were involved in enzyme assay. Y.A. wrote the first draft. T.F. initiated and supervised this study, reviewed and revised the manuscript, and approved the final version as submitted. All authors approved the final manuscript as submitted and agree to be accountable for all aspects of the work. All authors confirm the absence of previous similar or simultaneous publications.

## ETHICS APPROVAL

This study was approved by the Ethical Committee of the Graduate School of Medicine, Gifu University, Japan, and was carried out in accordance with the principles contained within the Declaration of Helsinki. Informed consents were obtained from the parents of the patient. No animals were used in the course of this research.
